# Evaluation of Nutritional, Phytochemical, and Mineral Composition of Selected Medicinal Plants for Therapeutic Uses from Cold Desert of Western Himalaya

**DOI:** 10.3390/plants10071429

**Published:** 2021-07-13

**Authors:** Radha, Manoj Kumar, Sunil Puri, Ashok Pundir, Sneh Punia Bangar, Sushil Changan, Poonam Choudhary, E. Parameswari, Ahmad Alhariri, Mahesh Kumar Samota, Rahul D. Damale, Surinder Singh, Mukesh K. Berwal, Sangram Dhumal, Anilkumar G. Bhoite, M. Senapathy, Anshu Sharma, Bharat Bhushan, Mohamed Mekhemar

**Affiliations:** 1 School of Biological and Environmental Sciences, Shoolini University of Biotechnology and Management Sciences, Solan 173229, India; sunilpuri@shooliniuniversity.com; 2 Chemical and Biochemical Processing Division, ICAR-Central Institute for Research on Cotton Technology, Mumbai 400019, India; 3 School of Mechanical and Civil Engineering, Shoolini University of Biotechnology and Management Sciences, Solan 173229, India; ashok.pundir78791@gmail.com; 4 Department of Food, Nutrition and Packaging Sciences, Clemson University, Clemson, SC 29634, USA; snehpunia69@gmail.com; 5 Division of Crop Physiology, Biochemistry and Post-Harvest Technology, ICAR—Central Potato Research Institute, Shimla 171001, India; sushil.changan@icar.gov.in; 6 Agricultural Structure and Environment Control, ICAR—Central Institute of Post-Harvest Engineering and Technology, Ludhiana 141004, India; poonam@icar.gov.in; 7 Department of Environmental Sciences, Tamil Nadu Agricultural University, Coimbatore 641003, India; parameswariphd@gmail.com; 8 Faculty of Agriculture, Damascus University, Damascus 30621, Syria; ahmadharere@yahoo.com; 9 HCP Division, ICAR—Central Institute of Post-Harvest Engineering and Technology, Abohar 152116, India; Mahesh.samota@icar.gov.in; 10 ICAR—National Research Centre on Pomegranate, Solapur 413255, India; rahul.damale@icar.gov.in; 11 Dr. S. S. Bhatnagar University Institute of Chemical Engineering and Technology, Panjab University, Chandigarh 160014, India; ssbhinder@pu.ac.in; 12 Division of Crop improvement, ICAR—Central Institute for Arid Horticulture, Bikaner 334006, India; mukesh.kumar4@icar.gov.in; 13 Division of Horticulture, RCSM College of Agriculture, Kolhapur 416004, India; sdhumal@msu.edu; 14 Department of Agricultural Botany, RCSM College of Agriculture, Kolhapur 416004, India; anilbhoite5001@gmail.com; 15 Department of Rural Development and Agricultural Extension, College of Agriculture, Wolaita Sodo University, Wolaita Sodo, Ethiopia; drsenapathy@wsu.edu.et; 16 Department of Food Science and Technology, Dr. Y.S. Parmar University of Horticulture and Forestry, Nauni 173230, India; anshufst1989@gmail.com; 17 ICAR—Indian Institute of Maize Research, Ludhiana 141004, India; bharat.bhushan@icar.gov.in; 18 Clinic for Conservative Dentistry and Periodontology, School of Dental Medicine, Christian-Albrecht’s University, 24105 Kiel, Germany

**Keywords:** elements, nutrition, medicinal plants, traditional knowledge

## Abstract

The aim of this study was to determine the elemental and nutritive values of leaf parts of 10 selected wild medicinal plants, *Acer pictum, Acer caecium, Betula utilis, Oxalis corniculata, Euphorbia pilosa, Heracleum lanatum, Urtica dioica, Berberis lycium*, *Berberis asiaticaand*, and *Quercus ilex,* collected from the high hills of the Chitkul range in district Kinnaur, Western Himalaya. The nutritional characteristics of medicinal plant species were analyzed by using muffle furnace and micro-Kjeldahl methods, and the mineral content in plants was analyzed through atomic absorption spectrometry. The highest percentage of used value was reported in *Betula utilis* (0.42) and the lowest in *Quercus ilex* (0.17). In this study, it was found that new generations are not much interested in traditional knowledge of ethnomedicinal plants due to modernization in society. Therefore, there is an urgent need to document ethnomedicinal plants along with their phytochemical and minerals analysis in study sites. It was found that rural people in western Himalaya are dependent on wild medicinal plants, and certain steps must be taken to conserve these plants from extinction in the cold desert of Himalayan region. They are an alternative source of medicine because they contain saponin, alkaloid, and flavonoid etc. as well as minerals. The leaves used for analysis possesses good mineral content, such as Na, N, K, P, Zn, Fe, Cu, Mn, Ca, Mg, and S. Hence, in the current study it was observed that medicinal plants are not only used for therapeutic purposes, but they can also be used as nutritional supplements.

## 1. Introduction

Plants are a good source of medicines and play a vital role in ethnic and tribal communities’ survival. Medicinal plants are used in all over the world to treat different types of human and animal diseases. The therapeutic usage of indigenous plant products for ethnomedicinal and nutritional objectives has attracted scientists’ curiosity, motivating them to look for bioactive compounds. Medicinal plants possess essential food components such as carbohydrates, protein, and fat. These components are important for the human body’s requirements and they are used in a different physiological, metabolic, and morphological activities [1,2]. Natural plant-derived products are used in medications, nutritional supplements, and in different healthcare products. Plants play a vital role in the identification of new beneficial medicinal components, and their phytochemical constituents such as antioxidants, hypoglycemic, and hypolipidemic constituents. Plants are often an excellent source of medicines, and many medicines are derived directly or indirectly from plant resources [3,4].

Plants are essential for daily life requirements of human beings, such as food, shelter, fiber, and therapeutic purposes. People in rural areas have preferred herbal medicines for health care. Sometimes in rural regions where conventional medicines are widely available, there has been an increase in interest in natural medications derived from plants and their use. Medicinal plants include a variety of physiologically active components, including minerals and phytochemicals, that show numerous physiological effects on humans [3,4]. The usage of essential oils is attracting significant attention, as these oils are a good source of various bioactive components. They are nowadays chosen over chemical antioxidants because of their antioxidant and antibacterial characteristics, and also due to their generally recognized as safe (GRAS) nature [5,6]. Essential oils are mostly made up of terpenes produced by the mevalonate pathway, which are complex combinations of volatile chemicals found in aromatic plants. Monoterpenes (hydrocarbon and oxygenated monoterpenes) and sesquiterpenes are among these volatile compounds (hydrocarbon and oxygenated sesquiterpenes). The medicinal and antimicrobial properties of angiosperm plants are used to treat constipation, dysentery, malaria, measles, onchocerciasis, stomach pain, yellow fever, and other ailments, while the plant’s slender roots and stem branches are used as munching sticks that are very effective in dental care. The analgesic activity, antibacterial activity, anticataleptic activity, antidiabetic and antihyperlipidemic activity, antifungal activity, antihypercholesterolemic activity, antimicrobial activity, antioxidant activity, diuretic, anti-inflammatory properties, hepatoprotective activity, and neuroprotective activity in Parkinson’s disease are all properties of Ginsenosides plants (Pinales). The determination of physiochemical and biological characteristics, sensory evaluation, and chemical composition analysis are all part of the entire characterization of an essential oil [7,8]. 

In recent years, there has been a significant increase in interest in botanical sources of natural pharmaceuticals, cosmetics, nutritional supplements, herbal teas, and other health-promoting items [9,10]. Throughout the world medicinal plants have been confirmed to contain essential bioactive compounds that can help to prevent different types of diseases such as cancer, heart disease, and diabetes. Medicinal plants serve a critical role in oral health diseases such as bleeding gums, mouth ulcers, dental caries, gingivitis, and halitosis since they have maximal efficiency or fewer side effects. Secondary metabolites are produced in different ways in different plant species [11,12,13]. Micronutrient and mineral deficiencies in the diet may have long-term negative effects on human health and lead to micronutrient deficiency diseases. Traditionally, all medicinal remedies were made from plants, whether in the form of a sample of plant parts or a more complex form such as crude extract combination. The primary advantage of using plant-derived drugs is that they are generally safer than synthetic substitutes, with significant therapeutic benefits and lower costs [14]. In different parts of India, there has been a growing interest in the study of medicinal plants and their traditional uses over the last few decades. Folk medicines have lost their value to the younger generation in recent years, and many young people are moving to urban areas for education and employment opportunities. Deforestation, environmental degradation, and modernization are currently occurring in the region, putting the country’s rich medicinal plant knowledge at risk. As a result, only the elderly have access to herbal knowledge, and only a small number of people are able to use conventional cures to treat illness. As a result, documenting indigenous people’s ancestral knowledge of traditional plant medicine will address the knowledge gap between ancestors and young folks on medicinal herbs [15].

The aim of this study was to determine the nutraceutical value and mineral elements of the 10 most commonly used ethnomedicinal plant species from the high hills of the Chitkul range. In the current study, 10 medicinal plants were selected for nutritional analysis because they were used for multiple purposes, such as foraging and edible uses (fruits, condiments, and vegetables). In order to prevent medicinal plant resources from becoming extinct, the current study recommends implementing various management strategies in collaboration with local communities, such as through village administrative councils. The most important aspect is to provide multi-directional awareness and training to local communities of the study region about sustainable plant exploitation. Tribal people reported that due to modernization in society, the new generation is not interested in traditional knowledge, so there is an urgent need to document this traditional knowledge from the study site of the high hills of the Chitkul range before it is lost to the society. As a result, it is essential to evaluate scientific documentation on plant parts collected from the cold desert of western Himalaya for the presence of necessary minerals and proximate composition in order to promote and scientifically confirm their health and ethnomedicinal advantages.

## 2. Results

Tribal people of the western Himalayan region are completely reliant on forest products to fulfill their daily life requirements such as food, fodder, fuel, shelter and medicines. The results of 10 selected ethnomedicinal plants used by tribal people in the high hills of the Chitkul range in the Kinnaur district were reported, and their nutritional and mineral values were analyzed.

### 2.1. Ethnobotanical Uses

Tribal people in the western Himalayan region of the high hills of the Chitkul range use 10 preferred ethnomedicinal plants which belong to the same or different families (Table 1). A total of 115 male informants were selected for interview and data collection. The tribal people reported that leaves were found to be the most frequently used part. In this study it was found that the different parts of plants used were found to be fruits (4), leaves (7), bark (1), seeds (1), stems (1), roots (4), and shoots (1).

The medicinal plants used in the study area were found to be *Acer pictum and A. caecium*, and their leaf and fruit extracts are applied to the skin for treating skin diseases; *Betula utilis* seeds and leaves are mixed with *Cynodon dactylon* and used on fractured parts of the body, and the fractured part of the body is covered with the bark of *Betula utilis;* fresh leaf juice of *Oxalis corniculata* is used for stomach infections; fresh stem and leaf juice of *Euphorbia pilosa* is used for jaundice; fresh root juice of *Heracleum lanatum* is taken internally for the treatment of cough and fever; fresh leaf, root, and shoot juice of *Urtica dioica* is used in the treatment of wound healing and skin allergies; *Berberis lycium* fruits are edible and highly nutritious for health and are used as a tonic, and root decoction is given for jaundice; *Quercus ilex* leaves are used for skin infection; and *Berberis asiatica* fruits are edible and highly nutritious for health and used as a tonic, and its roots are used in fever and asthma.

Tribal people reported that in the study area these 10 selected plants are easily available from the high hills to low hills of the western Himalayan region and these plants are highly effective for commonly found illnesses of human beings. Skin infections found in the study area include different types of diseses such as abscess, ringworm, acne, antiseptics, laceration, skin inflammation, blemishes, leucoderma, cuts and wounds, etc. The stomach disaeses found in the study region include stomach pain, constipation, dysentery, diarrhoea, and acitdity, etc. These plants are organized in a systematic manner, with botanical names, common names, families, parts used, routes, and ailments treated. Table 2 presents detailed information of these plants. In earlier studies from different parts of Himachal Pradesh it was reported that *Betula utilis* and *Berberis lycium* are used to treat different human ailments such as jaundice and wounds [14,15]. The Use value is a quantitative measure for the relative importance of species obtained by calculating the use value, where U is the number of use reports cited by each informant for a given species and n refers to the total number of informants. The highest percentage of used value was found in *Betula utiilis* (0.42) and lowest in *Quercus ilex* (0.17).

### 2.2. Nutrient Composition

The nutrient and phytochemical content were analyzed in 10 selected medicinal plant collected from the high hills of the Chitkul range. The determination of nutritional value in medicinal plants was investigated because they were used for multiple purposes: *Berberis aristata, B. lycium* fruits are edible, *Betula utilis* leaf extract is used in bone fracture and its bark is used as condiment, *Urtica dioica* leaves are used as a vegetable, and *Heracleum lanatum, Quercus ilex, Acer pictum, A. caecium, Berberis aristata, B. lycium, Oxalis corniculata, Urtica dioica* and *Euphorbia pilosa* are browsed by livestock in the study region. *Betula utilis* seeds, leaves and bark are used for skin infection (topically).

In the current study, moisture content was found to be highest in *Oxalis corniculata* (69.06%) and lowest in *Acer pictum* (57.95%); ash content was found highest in *Quercus ilex* (8.3%) and lowest in *Oxalis corniculata* (4.26%); crude fat was found highest in *Berberis asiatica* (2.53%) and lowest in *Euphorbia pilosa* (1.33%); crude fiber was highest in *Berberis lycium* (30.03%) and lowest in *Quercus ilex* (20.73%); crude protein was reported highest in *Acer pictum* (6.57%) and lowest in *Oxalis corniculata* (4.30%); carbohydrate was found highest in *Acer caecium* (27.92%) and lowest in *Oxalis corniculata* (21.10%); neutral detergent fiber was found highest in *Acer pictum* (59.34%) and lowest in *Euphorbia pilosa* (48.89%); acid detergent fiber was reported highest in *Acer pictum* (54.83%) and lowest in *Heracleum lanatum* (43.07%); alkaloid was found highest in *Quercus ilex* (0.80%) and lowest in *Berberis asiatica* (0.30%); flavanoid was found highest in *Berberis lycium* (0.82%) and lowest in *Acer caecium* (0.55%); and saponin was reported highest in *Heracleum lanatum* (2.84%) and lowest in *Acer pictum* (1.07%) (Table 2).

The quantity and quality of crude protein content found in plant samples are essential for the choice of plant species for nutraceutical significance, plant improvement programs, and systematic classification [16]. In earlier studies by different researchers, protein was determined using the Kjeldahl method. Protein, fats, and carbohydrates are the essential nutrients reported in several studies. Wild plant species are effective foundations of nutrition [17]. 

Plants are significant for providing energy and nutritional requirements to human beings. The carbohydrates, proteins, and fats are the nutrients found in plants, as well as minerals, play a vital role in creating a healthier organ control system in human beings [18]. The experimental plants will be characterized and standardized using the physicochemical parameters studied. The higher degree of food spoilage is mainly explained by a higher moisture content of in the leaves (75%). The ash content in a food material determines the consistency of the material, identifying it as carbon-free and showing the organic, inorganic, and impurity content found in the sample. The soluble and insoluble minerals in the sample are predicted by the total ash content [19]. In previous studies it was observed that leaf samples are high in carbohydrate content, which helps to retain energy potential, proteins, which are the building blocks of cells, fats, which provide energy and assist in the absorption of fat-soluble vitamins, and crude fiber, which helps in digestion [20].

### 2.3. Mineral Composition

The analysis for several micro- and macro-elements in the plants indicated that these were present in all plant samples which are responsible for curing different types of diseases. A variety of factors have been attributed to the increasing public interest in herbal remedies, some of which include the high cost and side effects of most modern medications [21]. In the present study, the highest percentage of Na was found in *Acer pictum* 45 μg/g and lowest in *Urtica dioica* 17 μg/g; N was found highest in *Acer pictum* (1.06%) and lowest in *Berberis asiatica* (0.42%); K was found highest in *Quercus ilex* 3.17% and lowest in *Urtica dioica* (1.54%); P was found highest in *Heracleum lanatum* (0.33%) and lowest in *Acer pictum* (0.12%). Zn was found highest in *Acer pictum* (58.71 μg/g) and lowest in *Oxalis corniculata* 18.03 μg/g; Fe was found highest in *Acer pictum* 322.38 μg/g and lowest in *Oxalis corniculata* 161.71 μg/g; Cu was found highest in *Acer caecium* 69.98 μg/g and lowest in *Oxalis corniculata* 34.32 μg/g; Mn was found highest in *Acer pictum* (87.08 μg/g) and lowest in *Urtica dioica* 46.54 μg/g; Ca was found highest in *Acer pictum* (77,105 μg/g) and lowest in *Urtica dioica* (32,987 μg/g); Mg was found highest in *Quercus ilex* (43,876 μg/g) and lowest in *Acer pictum* (17,792 μg/g); S was found highest in *Quercus ilex* (2587 μg/g) and lowest in *Acer caecium* (1498 μg/g); and Cr was found highest in *Heracleum lanatum* (6 μg/g) and lowest in *Oxalis corniculata* (2 μg/g). In different plant species, elemental accumulation depends on various factors such as the type of soil, fertilization method, plant species, and environmental circumstances [22]. The overall study indicated that medicinal plants are a significant source of mineral composition (Table 3).

According to earlier findings in the leaf samples of medicinal plants such as *Alternanthera sessilis*, the plant has higher content of Fe, Mn, Na, and K, moderate levels of Cu and Zn, and lower content of P. Similarly, in current studies it was found that all medicinal plants used for nutritional and minerals analysis have similar results to *Alternanthera sessilis*. Maintaining ionic equilibrium necessitates the use of K and Na. This connection between K and Na in foods helps to prevent hypertension [23,24]. In previous reports it was documented that Ca is essential for strong teeth and bones, as well as for structural rigidity to the body, and helps in blood clotting. Mn is required for enzymatic activities and is also essential for the formation of hemoglobin [25]. Cu is used in many enzymes as a structural constituent. Mg is important for energy metabolism, bone formation, and enzymatic action [26]. As compared to other minerals, Cr was found to be moderately poor. It plays a vital role, including the development of muscle and the maintenance of blood glucose in the body [27]. Zn acts as a stimulator in activating beta cells of the pancreas to release insulin and maintains a normal glucose rate. It is required for tissue repair and growth [28]. Fe is required for the production of hemoglobin, the transport of O_2_, and to increase the body’s immunity [29]. 

Therefore, it is essential to explore the traditional knowledge of the indigenous medicinal plants and to conduct pharmacological studies to find out their nutritive and therapeutic belonging [30,31,32]. The nutritional significance of plant species is measured by their content of carbohydrates, proteins, fats, oils, vitamins, minerals, and water, which are responsible for growth in flora and fauna species. The fats, protein, and carbohydrates are vital nutrients for life expectancy. The quantity and quality of proteins in the plants are important for the selection of plant species for nutritive significance [33,34]. Varying ecological circumstances can severely impact the quantities of essential plant elements. The ash percentage is a reflection of the mineral contents preserved in that plant species. The moisture percentage differs in plant species, dependent on the environment and physiology of the plants. Moisture percentage depends on the environmental circumstances such as temperature, humidity, harvest time, and type of weather as well as storage conditions.

## 3. Discussion

In this view, the study aimed to evaluate the nutritional, phytochemical, and mineral composition of selected medicinal plants for therapeutic uses from the cold desert of Western Himalaya. The presence of phytochemical ingredients that have a specific physiological action on the human body make these plants effective for both treating and curing human ailments. Medicinal plants are used to make pharmaceuticals either alone or in combination, and they can also be utilized as a source of raw materials for other medications [35]. Due to the fact that mineral elements make up a small amount of the total composition of most plant materials and of total body weight, they are extremely important physiologically, notably in the body’s metabolism. Apart from organic substances, it is now well documented that a few trace elements play an important role in overall health and disease prevention. Several investigations have found elemental content in plant extracts that we eat as herbal health supplements or medicine. These elements are found in different amounts in various sections of the plants, particularly in the roots, seeds, and leaves, which are consumed as well as utilized as a therapeutic substance [36,37]. Biochemical processes in the human body are affected by macro and trace elements. The metabolism is influenced by active ingredients of medicinal plants, such as metabolic products of plant cells and a number of mineral elements [38]. Some minerals are still chelated with organic ligands, making them accessible to the body [39]. Mineral element determination in plants is critical since the concentration and type of minerals affect the quality of many food items [40]. In this approach, not only must the absolute quantity of minerals in edible sections of plants be assessed, but these minerals must also be in bioavailable forms for the organism. Scientists and nutritionists have begun to believe in the physiological value of elements in animal health [41]. Minerals are needed by living organisms and can prevent the occurrence of certain ailments. Minerals are found in considerable amounts in some plants; their existence and quantity are determined by the plant’s family, history, and phytochemical characteristics [42]. The existence of a variety of phytochemical and elemental compositions contributes to the plant’s medicinal efficacy. As a result, it is critical to look into the phytoconstituents, components, and vitamin supplements found in medicinal plants in order to determine their therapeutic potential. Plants are a rich source of all of the components that humans require. Medicinal plants have been used in therapeutics and as nutritional supplements since before recorded history but have risen rapidly in recent years [43]. In the current study, ash, crude protein, and carbohydrate content in *Berberis asiatica* was found to be 6.33% 5.21%, and 17.36%; in *Beberis lycium* ash, crude protein, and carbohydrate content was found to be 5.40%, 5.84%, and 27.23%; but a study performed in the South Waziristan area of Pakistan on *Berberis lycium*, ash, crude protein, and carbohydrate content was found to be 7.75%, 7.67%, and 46.99%. Ash content was found highest in *Berberis lycium* at *7.75%* in the study performed in the South Waziristan area of Pakistan, but in our study ash content was found to be less, at 6.33%. It was observed that crude protein percentage was high in our study as compared to the study performed in the South Waziristan area of Pakistan but carbohydrate percentage was high in the study performed in the South Waziristan area of Pakistan as compared to our study. Overall study of the literature showed that the nutrient analysis was within the range of other studies. The marked difference from the other reported value could be associated with several attributes such as season and maturity stage of the foliage used for analysis [44]. Because ashing removes all of the organic material in the sample, ash contains inorganic plant components. Ash is also a sign of a plant’s high digestibility [45]. The presence of fiber is importance to human health because fibers are easily digested [46]. Dietary fiber is required for efficient digestion and waste removal. It has been shown to lower blood cholesterol, as well as the risk of coronary heart disease, hypertension, constipation, diabetes, and breast cancer [47]. The qualitative or quantitative measurement of mineral elements found in plants is essential. Mineral elements are also required in trace amounts for the efficient functioning of human systems, as well as for healthy development and growth [48]. The amount of mineral components in plants is highly dependent on soil abundance, especially fertility intensity [49]. Calcium is one of the minerals that are thought to play a role in fruit storage quality [50]. Calcium is the most abundant mineral in the bones and is required for a variety of cellular functions, including neuron and muscle function, hormone responses, blood clotting, and cellular death [51]. Calcium is required for the maintenance of healthy bones, teeth, and blood [52,53]. The most well-known element in the biological system is iron. It has a diverse set of biological roles. In the metabolic process, iron plays a particular role. Iron’s involvement in the body is firmly linked to hemoglobin and oxygen transmission from the lungs to tissue cells [54]. The most common dietary shortfall in humans is iron insufficiency [55]. Antimicrobial, antioxidant, anti-stress, and nutrigenomic effects on the development of immunity have made phytochemicals appealing for use as growth promoters in animal production [56]. In vitro and animal model studies, flavonoids have been shown to have anti-inflammatory, anticarcinogenic, antioxidant, antibacterial, antithrombotic, antiviral, hepatoprotective, and antiallergic effects [57]. According to previous research, a mixture of saponin-containing plants enriched with polyphenol improved weight gain when compared to those given antibiotics and growth promoters, implying that the saponin mixture could be used as an alternative to antibiotics and growth promoters in livestock production [58]. In earlier studies, different medicinal plants, such as *B. lyceum*, have been shown to contain a number of bioactive chemicals. Berberine, an alkaloid-like compound, has been identified as the most abundant among all *Berberis* species. Many researchers have reported on the isolation and characterization of berberine and other alkaloids such as jatrorrhizine, palmatine, berbamine, karakoramine, berbamunine, chenabine, and Sitosterol. Some phytochemicals found in *B. lyceum* fruit include anthocyanin, carotene, and ascorbic acid. The phytochemicals obtained from *Berberis* species are used to treat different ailments such as Jaundice [59]. In *Urtica dioica* the volatile compounds and the major ubiquitous primary metabolites, carbohydrates, aminoacids, organic acids and fatty acids have been identified in different parts. In addition, the secondary metabolites belonging to the classes of terpenoids, phenolics and flavonoids have been reported in addition to choline, the compound responsible for urticaria [60]. Sitosterol, betulin, betulic acid, oleanolic acid, acetyloleanolic acid, lupeol, lupenone, methyl betulonate, methyl betulate, and a novel triterpenoid karachic acid are all found in the bark of *Betula utilis*. This plant also contains leucocyanidin and polymeric leucoanthocyanidins [61]. In the northwestern Himalayan region, a high diversity of ethnobotanical plants are present, and as such, it is recommended that future phytochemical and mineral analysis should be performed on the plants species which have not been reported upon earlier from the study region. This study found that the leaves of the 10 selected plants are a good source of phytochemicals and minerals, suggesting that they could be a source of beneficial pharmaceuticals. Because these plants have a significant amount of readily available phyto-constituents and minerals, they can be used to replenish nutrients that are scarce or unavailable.

## 4. Material and Methods

### 4.1. Study Site

**Figure 1:**
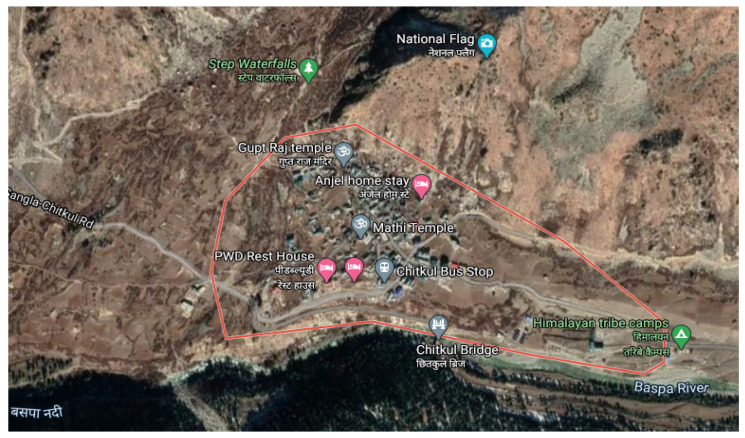
Satellite map showing study site.

The current survey was carried out in the high hills of the Chitkul range in the Kinnaur district of Himachal Pradesh, India. The tribal people of the the Chitkul range are dependent on forests for their daily requirements. Extensive field visits were undertaken in the study site to collect ethnobotanical data during 2017 to 2019 (Figure 1). During this period, the average temperature of the study site of the Kinnaur district ranges in the months of June and July was found to be between 16 to 20 °C.

### 4.2. Ethnobotanical Data Collection

The information on medicinal plants used to treat human diseases was collected through direct observations and interviews with elderly native people who have a wealth of traditional knowledge. The medicinal plant specimens collected from the high hills of Chitkul were identified from B.S.I. Dehradun, Uttrakhand. The tribal people of the study site collected plants based on their ethnomedicinal value. The phytochemical study was performed on 10 selected medicinal plants including *Acer pictum, A. caecium, Betula utilis, Oxalis corniculata, Euphorbia pilosa, Heracleum lanatum, Urtica dioica, Berberis lycium, Quercus ilex* and *Berberis asiatica* reported from the high hills of the Chitkul range. The proximate composition of minerals and nutrients in these plants was analyzed.

### 4.3. Determination of Nutrients

#### 4.3.1. Sample Preparation

The collected leaf samples were washed through 0.1% HgCl_2_ for several minutes and then dried. The dried samples were powdered and stored in pre-cleaned polyethylene bottles until the analysis started. The elemental analysis was performed at C.P.R.I, Shimla. Dried leaf in powder form was used for analysis through AAS. The nutritional analysis was performed at Shoolini University, Solan, Himachal Pradesh, India. The nutritional analysis of 10 selected ethnomedicinal plants from the high hills of the Chitkul range was expressed as mean ± SE of three replicates.

#### 4.3.2. Determination of the Moisture

A clean container was oven-dried at 105 °C for 1 h, and then cooled and weighed (W1). A dry leaf sample weighing 2 g (W2) was placed into the container and oven-dried at 105 °C. The sample was then cooled in a desiccator and weighed (W3) [59].

(1) $$\mathrm{Moisture}\;(\%)=\frac{W2-W3}{W2-W1}\times 100$$

#### 4.3.3. Determination of Ash

A porcelain crucible was dried at 105 °C for 1 h, and then cooled and weighed (W1). The 2 g of ground leaf sample was placed in the crucible, after which it was again weighed (W2). The crucible with plant samples was ashed first at 250 °C for an hour, followed by ashing at 550 °C for five hours in a muffle heating system. The leaf sample was then cooled in a desiccator and weighed again (W3) [59].

(2) $$\mathrm{Ash}\;(\%)=\frac{w2-w3}{w2-w1}\times 100$$

#### 4.3.4. Determination of Crude Fat

The 5g powdered sample was extracted in 100 mL diethyl ether and shaken it for 24 h in an orbital shaker. The ether extract was collected in a previously weighed beaker (W1). The filtrate was collected in the same beaker after it was equilibrated with 100 mL diethyl ether and again shaken for 24 h (W1). The ether was dried in an oven at 40–60 °C after being concentrated to dryness in a steam bath, and then the beaker was reweighed (W2) [62].

(3) $$\mathrm{Crude}\;\mathrm{fat}\;(\%)=\frac{\;\mathrm{Weight}\;\mathrm{of}\;\mathrm{flask}\;\mathrm{with}\;\mathrm{fat}\;-\;\mathrm{weight}\;\mathrm{of}\;\mathrm{empty}\;\mathrm{flask}}{\mathrm{Weight}\;\mathrm{of}\;\mathrm{original}\;\mathrm{sample}}\times 100$$

#### 4.3.5. Determination of Crude Fiber

A dried leaf sample (2 g) was processed with 100 mL of 1.25% H_2_SO_4_ for half an hour and filtered with pressure. The remaining residue was then washed with hot water. This process was repeated on the residue by using 100 mL of 1.25% NaOH sol. The remaining filtrate was dried at 100°C and weighed (C1). It was subsequently incinerated in a muffle furnace at 550 °C for 5 h and then reweighed (C2) [59].

(4) $$\mathrm{Crude}\;\mathrm{fiber}\;(\%)=\frac{C2-C1}{\mathrm{Weight}\;\mathrm{of}\;\mathrm{original}\;\mathrm{sample}}\times 100$$

#### 4.3.6. Determination of Crude Protein

Crude protein was determined by the Kjeldahl method [59].

(5) $$\mathrm{Crude}\;\mathrm{protein}\;(\%)=\frac{\left(\mathrm{mL}\;\mathrm{standard}\;\mathrm{acid}\;\times \;N\;\mathrm{of}\;\mathrm{acid}\right)\;-\;\left(\mathrm{mL}\;\mathrm{blank}\;\times \;N\;\mathrm{of}\;\mathrm{base}\right)\;-\;\left(\mathrm{mL}\;\mathrm{std}\;\mathrm{base}\;\times \;N\;\mathrm{of}\;\mathrm{base}\right)\times 1:4007}{\mathrm{Weight}\;\mathrm{of}\;\mathrm{sample}\;\mathrm{in}\;\mathrm{grams}}\times 100$$

where, N = normality, and percentage of crude protein was obtained by multiplying the nitrogen value by a factor of 6.25%. Crude protein = Nitrogen in sample × 6.25.

#### 4.3.7. Determination of Carbohydrate

The carbohydrate content was calculated by subtracting the total crude protein, crude fiber, ash, and lipid from the total dry matter [59]. 

Carbohydrate (%) = 100 − (% Moisture + % Ash + % Crude Fat + % Crude Fiber + % Crude Protein).

#### 4.3.8. Neutral Detergent Fiber

NDF was calculated using the following formula [60].

(6) $$\mathrm{NDF}\;(\%)=\frac{\left(\mathrm{Weight}\;\mathrm{of}\;\mathrm{crucible}+\mathrm{Fiber}\;\mathrm{content}\right)-\mathrm{Weight}\;\mathrm{of}\;\mathrm{empty}\;\mathrm{cricible}}{\mathrm{Weight}\;\mathrm{of}\;\mathrm{sample}}\times 100$$

#### 4.3.9. Acid Detergent Fiber

ADF was calculated using the following formula [60].

(7) $$\mathrm{ADF}\;(\%)=\frac{\mathrm{Weight}\;\mathrm{of}\;\mathrm{crucible}+\mathrm{Fiber}\;\mathrm{content}}{\mathrm{Weight}\;\mathrm{of}\;\mathrm{sample}}\times 100$$

### 4.4. Determination of Phytochemicals

#### 4.4.1. Alkaloid

A total of 5 g of dried leaf extract was weighed and mixed with 200 mL of 10% acetic acid in ethanol. The mixture was covered and left for 4 h. This mixture was filtered and the filtrate was concentrated on a water bath to a quarter of the initial volume. The concentrated NH_4_OH was added to the extract until precipitation was completed. The solution was then washed with dilute NH_4_OH and filtered. The residue collected was dried and weighed and the alkaloid content was determined [59].

(8) $$\mathrm{Alkaloid}\;(\%)=\frac{\mathrm{Weight}\;\mathrm{of}\;\mathrm{precipitate}}{\mathrm{Weight}\;\mathrm{of}\;\mathrm{original}\;\mathrm{sample}}\times 100$$

#### 4.4.2. Flavonoid

The 10 g leaf plant powder was extracted repetitively with 100 mL of 80% aqueous methanol each for 3 days. The whole solution was later filtered through Whatman filter paper. The obtained filtrate was then transferred into a crucible and evaporated to dryness in a water bath and weighed to a constant weight. The weight obtained gave the estimation of flavonoids content in the plant leaf sample [61].

(9) $$\mathrm{Flavonoid}\;(\%)\;\frac{\mathrm{Weight}\;\mathrm{of}\;\mathrm{dried}\;\mathrm{sample}}{\mathrm{Weight}\;\mathrm{of}\;\mathrm{original}\;\mathrm{sample}\times 1}\times 100$$

#### 4.4.3. Saponin

The 5 g of dried leaf powder was added to 50 mL of 20% ethanol, kept on a shaker for 30 min and then heated in a water bath at 55 °C for 4 h. The mixture was filtered through Whatman filter paper. The collected residue was re-extracted with another 200 mL of 20% aq. ethanol. The filtrates were combined and reduced to 40 mL in a water bath at 90 °C. The concentrate was transferred into a separating funnel, 20 mL of diethyl ether (C_2_H_5_)_2_O was added, and strongly shaken. The ether layer, which was the upper layer, was discarded and the aqueous layer was retained in a beaker. The retained layer was re-introduced into a separating funnel and 60 mL of n-butanol was added and vigorously shaken. The C₄H₁₀O extract upper layer was retained while the bottom layer was discarded. The C₄H₁₀O layer was washed twice with 10 mL of 5% aqueous sodium chloride. The remaining solution was collected and heated to evaporation in a water bath, then dried to constant weight at 40 °C in an oven [59].

(10) $$\mathrm{Saponin}\;(\%)=\frac{\mathrm{Weight}\;\mathrm{of}\;\mathrm{residue}}{\mathrm{Weight}\;\mathrm{of}\;\mathrm{original}\;\mathrm{sample}}\times 100$$

### 4.5. Determination of Minerals (N, P, K, Ca, Cu, Fe, Zn, Na, Cr, Mn, Mg, and S)

#### 4.5.1. Sample Collection and Preparation

The collected leaf samples were washed with deionized water and then spread on a sheet of paper for air drying. The dried samples were crushed and stored in a dry and dark place at room temperature in polythene bags for further mineral analysis.

#### 4.5.2. Mineral Analysis

In the present study, nutrient analysis of P, K, Ca, Cu, Fe, Zn, Na, Cr, Mn, Mg, and S in leaf samples was analyzed through AAS after digestion of the leaf sample [62].Nitrogen in different leaf samples was analyzed through the Kjeldahl method [62]. 

### 4.6. Statistical Analysis

Data was examined with SPSS Version 20.0. Results were determined as Mean ± SE of three repeat determinations.

## 5. Conclusions

The study revealed that medicinal plants in the study area are rich in phytochemicals and mineral content. The medicinal plants have a wide range of potential applications in the manufacture of new drugs, nutraceuticals, and healthcare products. The medicinal plants comprise a variety of pharmacologically active phytochemicals that have been used to treat different types of diseases. These medicinal plant species are used to treat both minor illnesses (cough, cold, fever, and skin infections) and major illnesses (asthma and jaundice). The ability to determine element concentrations in plants offers many possibilities for therapeutic applications. In the study region, rural people have significant traditional knowledge of medicinal plants for treating a variety of human and animal disease. During interaction with tribal people, they reported that the new generation is not much interested in traditional knowledge due to modernization in society, so proper documentation of medicinal plants is needed from the study site. Further research should be carried out in order to describe the photochemistry that may be helpful for new drug development.

## Figures and Tables

**Table 1:** Ethnomedicinal plants used by tribal people in the study site.

Sr. No.	Botanical Name	Family	Ethnobotanical Origin	Habit	Common Name	Pats Used	Flowering and Fruiting Months	Routes of Administration	Ailment Treated	Used Value
**1**	*Acer pictum* Thunb.	Sapindaceae	Korea, Japan, Mongolia, Russia	Tree	Kandal	Leaves, Fruits	April-October	Topical	Leaf and fruit extracts are applied to the skin.	0.24
**2**	*Acer caecium* Wall. Ex D.Don	Sapindaceae	Pakistan, India, Nepal, China	Tree	Kandal	Leaves, Fruits	April-October	Topical	Leaf and fruits extracts are applied to the skin.	0.21
**3**	*Betula utilis* D.Don	Betulaceae	India, Bhutan, Afghanistan	Tree	Bhojpatra	Seeds, Leaves, Bark	May-October	Topical	Seeds and leaves are mixed with *Cynodon dactylon* and a paste prepared and used on fractured parts of the body, which are then covered with the bark of *Betula utilis*. Bark extract is used in wounds.	0.42
**4**	*Oxalis corniculata* L.	Oxalidaceae	America, India, Mexico	Herb	Amrul	Leaves	April-October	Oral	Fresh juice of fresh leaves is used for stomach infections.	0.33
**5**	*Euphorbia pilosa* L.	Euphorbiaceae	India, Siberia, Mongolia	Herb	Duddhi	Stem, Leaves	November-April	Oral	Fresh juice of stem and leaves is used for jaundice.	0.36
**6**	*Heracleum lanatum* Michx.	Apiaceae	India, America, Bangladesh, China, Nepal, Israel, Bhutan	Herb	Patrala	Roots	June-July	Oral	Fresh juice of roots is taken internally for the treatment of cough and fever.	0.34
**7**	*Urtica dioica* L.	Urticaceae	Africa, Australia, America, India	Herb	Bichhu Bootii	Leaves, Roots, Shoots	June-October	Oral	Fresh juices of leaves, roots, and shoots are used for wound healing and skin allergy.	0.26
**8**	*Berberis lycium* Royle	Berberidaceae	India, Nepal, Afghanistan, Bangladesh, Pakistan	Shrub	Kashmal	Fruits, Roots	March-July	Oral	Fruits are used as a tonic. Roots decoction is given for jaundice.	0.39
**9**	*Quercus ilex* L.	Fagaceae	India, Germany, Italy, Spain, Austria, Greece, Netherland	Shrub	-	Leaves	September- October	Topical	Leaves are used for skin allergies.	0.17
**10**	*Berberis asiatica* Roxb. ex. DC	Berberidaceae	Asia, Europe, America	Shrub	Kashmal	Fruits, Roots	March-July	Oral	Fruits are edible and highly nutritious for health, so are used as a tonic. Root decoction is given for fever and asthma.	0.40

**Table 2:** Proximate and phytochemical composition of medicinal plants’ leaves collected from study site. Individual readings (*n* = 3) were averaged and are presented with ±SE.

Sr. No.	Species Name	Moisture%	Ash %	Crude Fat %	Crude Fiber %	Crude Protein %	Carbohydrate%	NDF %	ADF %	Alkaloid %	Flavanoid%	Saponin%
1	*Acer pictum* Thunb.	57.95 ± 0.31	6.13 ± 0.23	1.97 ± 0.06	27.63 ± 0.42	6.57 ± 0.05	27.82 ± 0.34	59.34 ± 0.36	54.83 ± 0.41	0.56 ± 0.03	0.74 ± 0.02	1.07 ± 0.06
2	*Acer caecium* Wall. Ex D.Don	58.2 ± 0.43	6.93 ± 0.17	2.28 ± 0.09	26.93 ± 0.33	4.99 ± 0.07	27.92 ± 0.29	57.44 ± 0.38	49.61 ± 0.35	0.39 ± 0.02	0.55 ± 0.02	1.85 ± 0.08
3	*Betula utilis* D.Don	65.33 ± 0.32	5.53 ± 0.38	2.2 ± 0.08	26.83 ± 0.43	5.29 ± 0.05	22.05 ± 0.28	58.48 ± 0.4	47.77 ± 0.37	0.53 ± 0.02	0.7 ± 0.01	1.53 ± 0.06
4	*Oxalis corniculata* L.	69.06 ± 0.41	4.26 ± 0.31	1.42 ± 0.08	29.96 ± 0.27	4.3 ± 0.08	21.1 ± 0.28	53.51 ± 0.32	47.97 ± 0.43	0.36 ± 0.02	0.62 ± 0.02	1.92 ± 0.08
5	*Euphorbia pilosa* L.	62.84 ± 0.41	4.6 ± 0.34	1.33 ± 0.06	20.86 ± 0.49	4.34 ± 0.06	27.09 ± 0.26	48.89 ± 0.35	44.87 ± 0.42	0.52 ± 0.02	0.69 ± 0.02	2.42 ± 0.09
6	*Heracleum lanatum* Michx.	65.03 ± 0.29	5.3 ± 0.26	1.47 ± 0.06	25.06 ± 0.34	3.38 ± 0.07	27.13 ± 0.26	49.64 ± 0.27	43.07 ± 0.5	0.73 ± 0.02	0.76 ± 0.02	2.84 ± 0.05
7	*Urtica dioica* L.	61.83 ± 0.37	4.73 ± 0.32	1.37 ± 0.05	26.06 ± 0.34	4.74 ± 0.04	24.47 ± 0.28	48.95 ± 0.33	43.24 ± 0.37	0.42 ± 0.03	0.65 ± 0.02	2.32 ± 0.06
8	*Berberis lycium* Royle	64.66 ± 0.43	6.33 ± 0.26	2.24 ± 0.06	30.03 ± 0.24	5.84 ± 0.05	27.23 ± 0.24	57.92 ± 0.44	49.16 ± 0.41	0.67 ± 0.02	0.82 ± 0.02	1.99 ± 0.07
9	*Quercus ilex* L.	66.78 ± 0.47	8.3 ± 0.2	1.59 ± 0.09	20.73 ± 0.33	5.74 ± 0.06	21.38 ± 0.31	56.96 ± 0.43	53.53 ± 0.36	0.8 ± 0.02	0.59 ± 0.02	1.97 ± 0.09
10	*Berberis asiatica* Roxb. ex. DC	65.3 ± 0.43	5.4 ± 0.32	2.53 ± 0.04	25.23 ± 0.33	5.28 ± 0.1	17.86 ± 0.22	54.94 ± 0.45	52.1 ± 0.35	0.3 ± 0.02	0.56 ± 0.02	2.82 ± 0.07

**Table 3:** Mineral’s composition in some selected foliage leaves from the study site.

Sr. No.	Fodder Species	Na μg/g	N %	K %	P%	Zn (μg/g)	Fe (μg/g)	Cu (μg/g)	Mn (μg/g)	Ca (μg/g)	Mg (μg/g)	S (μg/g)	Cr (μg/g)
1	*Acer pictum* Thunb.	45	1.06	1.87	0.12	58.71	322.38	64.67	87.08	77,105	17,792	1576	3
2	*Acer caecium* Wall. Ex D.Don	40	0.98	2.03	0.19	45.78	228.52	69.98	72.98	70,314	20,987	1498	3
3	*Betula utilis* D.Don	37	0.84	2.23	0.2	42.83	241.7	59.67	76.67	72,567	23,276	2187	3
4	*Oxalis corniculata* L.	20	0.67	1.87	0.21	18.03	161.71	34.32	56.55	67,347	17,865	2398	2
5	*Euphorbia pilosa* L.	23	0.71	1.56	0.3	28.89	179.08	40.43	52.78	64,675	25,897	1789	3
6	*Heracleum lanatum* Michx.	25	0.56	2.32	0.33	34.56	215.97	46.76	61.34	60,542	31,778	2198	6
7	*Urtica dioica* L.	17	0.77	1.54	0.21	45.87	194.65	41.98	46.54	32,987	29,832	2487	3
8	*Berberis lycium* Royle	21	0.81	2.15	0.2	49.34	189	58.87	54.87	39,876	20,651	2298	3
9	*Quercus ilex* L.	24	0.47	3.17	0.22	54.78	248.04	54.45	61.56	41,768	43,876	2587	5
10	*Berberis asiatica* Roxb. ex. DC	19	0.42	2.34	0.26	50.23	182.02	50.1	56.98	43,988	23,456	2476	3

## Data Availability

Not applicable.

## References

[1] Radha R., Chauhan P., Puri S., Sharma A.K., Pundir A. (2021). A study of wild medicinal plants used in Nargu Wildlife Sanctuary of district Mandi in Himachal Pradesh, India. J. Appl. Pharm. Sci..

[2] Cheeke P. Applications of saponins as feed additives in poultry production. Proceedings of the 20th Australian Poultry Science Symposium.

[3] Petrovska B.B. (2012). Historical review of medicinal plants’ usage. Pharmacogn. Rev..

[4] Dagli N., Dagli R., Mahmoud R.S., Baroudi K. (2015). Essential oils, their therapeutic properties, and implication in dentistry: A review. J. Int. Soc. Prev. Community Dent..

[5] Kumar M., Changan S., Tomar M., Prajapati U., Saurabh V., Hasan M., Sasi M., Maheshwari C., Singh S., Dhumal S. (2021). Custard Apple (*Annona squamosa* L.) Leaves: Nutritional Composition, Phytochemical Profile, and Health-Promoting Biological Activities. Biomolecules.

[6] Radha P.S., Chandel K., Pundir A., Thakur M.S., Chauhan B., Simer K., Dhiman N., Shivani T.Y.S., Kumar S. (2019). Diversity of ethnomedicinal plants in Churdhar Wildlife Sanctuary of district Sirmour of Himachal Pradesh, India. J. Appl. Pharm. Sci..

[7] Butnariu M., Sarac L. (2018). Essential oils from plants. J. Biotechnol. Biomed. Sci..

[8] Mekhemar M., Geib M., Kumar M., Radha, Hassan Y., Dörfer C. (2021). Salvadora persica: Nature’s Gift for Periodontal Health. Antioxidants.

[9] Rajurkar N., Damame M. (1997). Elemental analysis of some herbal plants used in the treatment of cardiovascular diseases by NAA and AAS. J. Radioanal. Nucl. Chem..

[10] Kumar M., Tomar M., Punia S., Grasso S., Arrutia F., Choudhary J., Singh S., Verma P., Mahapatra A., Patil S. (2021). Cottonseed: A sustainable contributor to global protein requirements. Trends Food Sci. Technol..

[11] Pandey D.K., Dey A. (2016). A validated and densitometric HPTLC method for the simultaneous quantification of reserpine and ajmalicine in Rauvolfia serpentina and Rauvolfia tetraphylla. Rev. Bras. Farm..

[12] Kumar M., Prakash S., Kumari N., Pundir A., Punia S., Saurabh V., Choudhary P., Changan S., Dhumal S., Pradhan P.C. (2021). Beneficial Role of Antioxidant Secondary Metabolites from Medicinal Plants in Maintaining Oral Health. Antioxidants.

[13] Ferrentino G., Morozova K., Horn C., Scampicchio M. (2020). Extraction of Essential Oils from Medicinal Plants and their Utilization as Food Antioxidants. Curr. Pharm. Des..

[14] Radha S.P., Pundir A. (2019). Survey of ethnomedicinal plants used by migratory shepherds in Shimla district of Himachal Pradesh. Plant Arch..

[15] Chauhan N.S. (1999). Medicinal and Aromatic Plants of Himachal Pradesh.

[16] Nisar T., Wang Z.-C., Yang X., Tian Y., Iqbal M., Guo Y. (2018). Characterization of citrus pectin films integrated with clove bud essential oil: Physical, thermal, barrier, antioxidant and antibacterial properties. Int. J. Biol. Macromol..

[17] Heywood V.H. (2011). Ethnopharmacology, food production, nutrition and biodiversity conservation: Towards a sustainable future for indigenous peoples. J. Ethnopharmacol..

[18] Rahmatollah R., Mahbobeh R. (2010). Mineral contents of some plants used in Iran. Pharmacogn. Res..

[19] Ilodibia C.V., Ewere F.U., Akachukwu E.E., Adimonyemma R.N., Igboabuchi N.A., Okeke N.F. (2016). Proximate Composition, Vitamin and Anatomical Studies on Gomphrena celosioides. Annu. Res. Rev. Biol..

[20] Aberoumand A. (2011). Protein, Fat, Calories, Minerals, Phytic acid and Phenolic in Some Plant Foods Based Diet. J. Food Process. Technol..

[21] Ahmad K. (2007). Antiretroviral therapy abandoned for herbal remedies. Lancet Infect. Dis..

[22] Bengtsson H., Öborn I., Jonsson S., Nilsson I., Andersson A. (2003). Field balances of some mineral nutrients and trace elements in organic and conventional dairy farming—a case study at Öjebyn, Sweden. Eur. J. Agron..

[23] Seal T. (2011). Determination of nutritive value, mineral contents and antioxidant activity of some wild edible plants from Meghalaya state, India. Asian J. Appl. Sci..

[24] Saupi N., Zakaria M.H., Bujang J.S. (2009). Analytical chemical composition and mineral content of yellow velvetleaf (Limnocharis flava L. Buchenau’s) edible parts. J. Appl. Sci..

[25] Indrayan A.K., Sharma S., Durgapal D., Kumar N., Kumar M. (2005). Determination of nutritive value and analysis of mineral elements for some medicinally valued plants from Uttaranchal. Curr. Sci..

[26] Smith J.C. (1987). Copper nutritive and cardiovascular integrity in: Hemophil DD. Proceedings of the 21st Annual Conference on Trace Substances in Environmental Health.

[27] Smith W.D., Hammarsten J.F. (1958). Serum Mg in clinical disorders. South Mol. J..

[28] Hambridge K.M. (1974). Chromium nutrition in man. Am. J. Clin. Nutr..

[29] Glew R., VanderJagt D., Bosse R., Huang Y.-S., Chuang L.-T. (2005). The nutrient content of three edible plants of the Republic of Niger. J. Food Compos. Anal..

[30] Albuquerque U.P., De Medeiros P.M., Ramos M.A., Júnior W.S.F., Nascimento A.L.B., Avilez W.M.T., De Melo J.G. (2014). Are ethnopharmacological surveys useful for the discovery and development of drugs from medicinal plants?. Rev. Bras. Farm..

[31] Chandra S., Rawat D. (2015). Medicinal plants of the family Caryophyllaceae: A review of ethno-medicinal uses and pharmacological properties. Integr. Med. Res..

[32] Uprety Y., Asselin H., Boon E.K., Yadav U., Shrestha K.K. (2010). Indigenous use and bio-efficacy of medicinal plants in the Rasuwa District, Central Nepal. J. Ethnobiol. Ethnomed..

[33] Day L. (2013). Proteins from land plants—Potential resources for human nutrition and food security. Trends Food Sci. Technol..

[34] Prajna P.S., Rama Bhat P. (2015). Phytochemical and mineral analysis of root of Loeseneriella arnottiana Wight. Int. J. Curr. Res. Biosci. Plant Biol..

[35] Radha S.P., Pundir A. (2019). Review on Ethnomedicinal Plant: Trillium govanianum Wall. Ex D. Don. Int. J. Theor. Appl. Sci..

[36] Van Soest P.J., Wine R.H. (1967). Use of Detergents in the Analysis of Fibrous Feeds. IV. Determination of Plant Cell-Wall Constituents. J. Assoc. Off. Anal. Chem..

[37] Boham B.A., Kocipai-Abyazan R. (1974). Flavonoids and condensed tannins from leaves of Hawaian vaccinium vaticulatum and V. calycinium. Pac. Sci..

[38] Dhyan S., Chhonkar P.K., Dwivedi B.S. (2005). Manual on Soil, Plant and Water Analysis.

[39] Mohanta B., Chakraborty A., Sudarshan M., Dutta R.K., Baruah M. (2003). Elemental profile in some common medicinal plants of India. Its correlation with traditional therapeutic usage. J. Radioanal. Nucl. Chem..

[40] Underwood E.J. (1977). Trace Elements in Human and Animal Nutrition.

[41] Prasad A.S. (1993). Essential and Toxic Elements in Human Health and Disease: An Update.

[42] Lokhande R., Singare P., Andhale M. (2010). Study on Mineral content of Some Ayurvedic Indian Medicinal Plants by Instrumental Neutron Activation Analysis and AAS Techniques. Health Sci. J..

[43] Kolasani A., Xu H., Millikan M. (2011). Evaluation of mineral content of Chinese medicinal herbs used to improve kidney function with chemometrics. Food Chem..

[44] Choudhury R.P., Garg A. (2007). Variation in essential, trace and toxic elemental contents in Murraya koenigii—A spice and medicinal herb from different Indian states. Food Chem..

[45] Ullah Z., Baloch M.K., Khader J.A., AbdEIslam N.M., Noor S. (2013). Proximate and nutrient analysis of selected medicinal plants of Tank and South Waziristan area of Pakistan. Afr. J. Pharm. Pharmacol..

[46] Udayakumar R., Begum V.H. (2004). Elemental analysis of Medicinal Plants used in controlling infectious diseases. Hamdard Med..

[47] Houghton P., Yanniv Z., Bachrach U. (2007). Use of medicinal plants in CNS disorders. Handbook of Medicinal Plants.

[48] Woods P.W. (1999). Herbal healing. Essence.

[49] Khan I.A., Allgood J., Walker L.A., Abourashed E.A., Schlenk D., Benson W.H. (2001). Determination of Heavy Metals and Pesticides in Ginseng Products. J. AOAC Int..

[50] Ibrahim J., Ajaegbu V.C., Egharevba H.O. (2010). Pharmacognostic and phytochemical analysis of *Commelina benghalensis* L.. Ethnobot. Leafl..

[51] Hussain J., Rehman N., Al-Harrasi A., Ali L., Ullah R., Mabood F., Hussain H., Ismail M. (2011). Nutritional prospects and mineral compositions of selected vegetables from Dhoda sharif Kohat. J. Med. Plants Res..

[52] Hussain J., Khan A.L., Rehman N., Khan F., Hu S.T., Shinw Z.K. (2009). Proximate and Nutrient Investigations of Selected Medicinal Plants Species of Pakistan. Pak. J. Nutr..

[53] Igwenyi I.O., Agwor A.S., Nwigboji I.U., Agbafor K.N., Offor C.E. (2014). Proximate Analysis, Mineral and Phytochemical Composition of Euphorbia Hyssopifolia. IOSR J. Dent. Med Sci..

[54] Kruczek A. (2005). Effect of row fertilization with different kinds of fertilizers on the maize yield. Acta Sci. Pol. Agric..

[55] Léchaudel M., Joas J., Caro Y., Génard M., Jannoyer M. (2004). Leaf:fruit ratio and irrigation supply affect seasonal changes in minerals, organic acids and sugars of mango fruit. J. Sci. Food Agric..

[56] Yagi S., Rahman A.E., ELhassan G.O., Mohammed A.M. (2013). Mohammed. Elemental analysis of ten Sudanese medicinal plants using X-ray fluorescence. J. Appl. Ind. Sci..

[57] Charles P. (1992). Calcium absorption and calcium bioavailability. J. Intern. Med..

[58] Hughes M.N. (1972). The Inorganic Chemistry of Biological Processes.

[59] Sigel H. (1978). Metals in Biological Systems.

[60] Nazir N., Rahman A., Uddin F., Khan Khalil A.A., Zahoor M., Nisar M., Ullah S., Ullah R., Ezzeldin E., Mostafa G.A. (2021). Quantitative Ethnomedicinal Status and Phytochemical Analysis of Berberis lyceum Royle. Agronomy..

[61] Grauso L., de Falco B., Lanzotti V., Motti R. (2020). Stinging nettle, *Urtica dioica* L.: Botanical, phytochemical and pharmacological overview. Phytochem. Rev..

[62] Adamu H.M., Ushie O.A., Gwangwala A.H., Yadav R.P., Singh A., Bhardwaj A.K., Lone P.A., Dar M.M., Parray J.A., Shah K.W. (2013). Estimation of Total Flavonoids and Tannins in the Stem Bark and Leaves of *Anogeisus leiocarpus* Plant plant. Int. J. Tradit. Nat. Med..

